# Consumption Trends of Antifungal and Antiprotozoal Agents for Human Systemic Use in Kazakhstan from 2017 to 2023

**DOI:** 10.3390/antibiotics13090857

**Published:** 2024-09-06

**Authors:** Yuliya Semenova, Assiya Kussainova, Laura Kassym, Ainur Aimurziyeva, Daniil Semenov, Lisa Lim

**Affiliations:** 1 School of Medicine, Nazarbayev University, Astana 010000, Kazakhstan; yuliya.semenova@nu.edu.kz (Y.S.); laura.kassym@nu.edu.kz (L.K.); 2 Department of General Practice with a Course of Evidence-Based Medicine, NJSC “Astana Medical University”, Astana 010000, Kazakhstan; 3 School of Sciences and Humanities, Nazarbayev University, Astana 010000, Kazakhstan; ainur.aimurziyeva@nu.edu.kz; 4 Computer Science and Engineering Program, Astana IT University, Astana 010000, Kazakhstan; daniil.semenov@nu.edu.kz; 5 Graduate School of Public Policy, Nazarbayev University, Astana 010000, Kazakhstan; lisa.lim@nu.edu.kz

**Keywords:** antifungal agents, antiprotozoal agents, consumption, antimicrobial stewardship, time series, Kazakhstan

## Abstract

Background/Objectives: While multiple studies have investigated antibiotic consumption rates, there are few studies on the consumption of systemic antifungals and antiprotozoals. This study aims to fill this gap by providing a comprehensive analysis of nationwide consumption trends in Kazakhstan over a seven-year period (2017–2023). Methods: Defined daily doses per 1000 inhabitants per day were calculated for systemic antifungals (J02 code of the Anatomical Therapeutic Chemical Classification System (ATC)) and antiprotozoals (P01 code of the ATC). Time series analyses were applied to examine historical trends, evaluate the impact of the COVID-19 pandemic, and make future projections until 2030. Results: The total consumption increased over the study period, with an average annual percent change of 1.11% for antifungals and 5.48% for antiprotozoals. Fluconazole was the most consumed antifungal agent, whereas metronidazole was the most consumed antiprotozoal agent. The COVID-19 pandemic had a positive but insignificant effect on the consumption of antifungals and a negative and also insignificant effect on the consumption of antiprotozoals. Forecast modeling indicates that the future trends in antifungal and antiprotozoal consumption until 2030 will largely remain stable, with the exception of antiprotozoal consumption in the hospital sector, which is projected to decline. Conclusions: These findings offer valuable insights into the development and implementation of targeted antimicrobial stewardship programs in Kazakhstan.

## 1. Introduction

Fungal and protozoal diseases represent significant public health threats worldwide due to their substantial morbidity and mortality rates, particularly among vulnerable populations such as immunocompromised individuals, elderly individuals, and children [1]. Fungal infections, such as candidiasis, aspergillosis, and cryptococcosis, are pervasive in both hospital and community settings [2]. These infections can lead to severe complications, especially in cancer patients, organ transplant recipients, patients with respiratory diseases, and those with HIV/AIDS [1,2]. Protozoal diseases, including malaria, leishmaniasis, and Chagas disease, predominantly affect tropical and subtropical regions but also pose risks globally due to increased travel and climate change [3]. Malaria alone accounts for hundreds of thousands of deaths annually, with children under five being particularly susceptible [4]. Leishmaniasis and Chagas disease, though less known, cause significant morbidity, including severe skin ulcers and organ damage, and, if left untreated, can be fatal [3].

Owing to various factors, low- and middle-income countries (LMICs) are particularly vulnerable to the impact of fungal and protozoal diseases, including limited access to healthcare, inadequate diagnostic facilities, and insufficient public health infrastructure. These regions often face challenges such as poor sanitation, limited availability of antifungal and antiprotozoal medications, and a lack of awareness of preventive measures [5]. The emergence of resistance to these medications further complicates treatment efforts, making it difficult to manage infections effectively [6]. Preparedness for future outbreaks is another concern, necessitating that public health authorities understand what antifungal and antiprotozoal medications are available, what their consumption rates are, and how to ensure their effective use [7].

The Republic of Kazakhstan (hereafter referred to as Kazakhstan) is a country in Central Asia that gained its independence in 1991 following the dissolution of the Soviet Union. The country is classified as upper–middle income but has an underfunded healthcare system, a legacy of the Semashko model [8]. Although over-the-counter (OTC) sales of antimicrobials are illegal and the country has implemented an antimicrobial stewardship (AMS) action plan since 2019, this initiative does not specifically address other categories of antimicrobial agents in addition to antibiotics [9]. While various studies have investigated antibiotic consumption in Kazakhstan [10,11], there is a gap in the research concerning the consumption of antifungals and antiprotozoals.

This study aims to fill this gap by investigating the consumption patterns of systemic antifungals and antiprotozoals over a period of seven years (from 2017 to 2023). Specifically, the study analyzes the trends in drug usage overall, as well as in the community and hospital sectors. In addition, this study assesses the impact of the COVID-19 pandemic on the consumption of these medications and provides future projections until 2030.

## 2. Results

Table 1 presents information on the total consumption of systemic antifungals and antiprotozoals across both the community and hospital sectors combined. The Anatomical Therapeutic Chemical Classification, level 5 (ATC5), is utilized to categorize specific drugs within these groups and to provide a detailed breakdown of consumption trends. Overall, from 2017 to 2023, the total consumption of antifungals (J02) and antiprotozoals (P01) tended to increase, with average annual percent changes (AAPCs) of 1.11% and 5.48%, respectively. A statistically significant change in AAPC was observed for the consumption of secnidazole (AAPC = −7.93%, *p* = 0.005). Fluconazole was the most consumed antifungal agent throughout the study period, except in 2021, when ketoconazole was the most consumed antifungal agent.

At the community level, the consumption of systemic antifungals and antiprotozoals also tended to increase, with AAPCs of 1.08% and 7.37%, respectively. A statistically significant negative trend was observed for the consumption of secnidazole (AAPC = −7.92%, *p* = 0.005). Like overall consumption, fluconazole was the most commonly consumed systemic antifungal throughout the entire period under study, except for 2021, when ketoconazole took over (Table 2).

In the hospital sector, the consumption of systemic antifungals increased (AAPC = 1.14%), whereas the consumption of systemic antiprotozoals decreased (AAPC = −27.59%). Fluconazole was also the most consumed systemic antifungal, except in 2021, when ketoconazole was the most consumed antifungal (Table 3).

**Figure 1:**
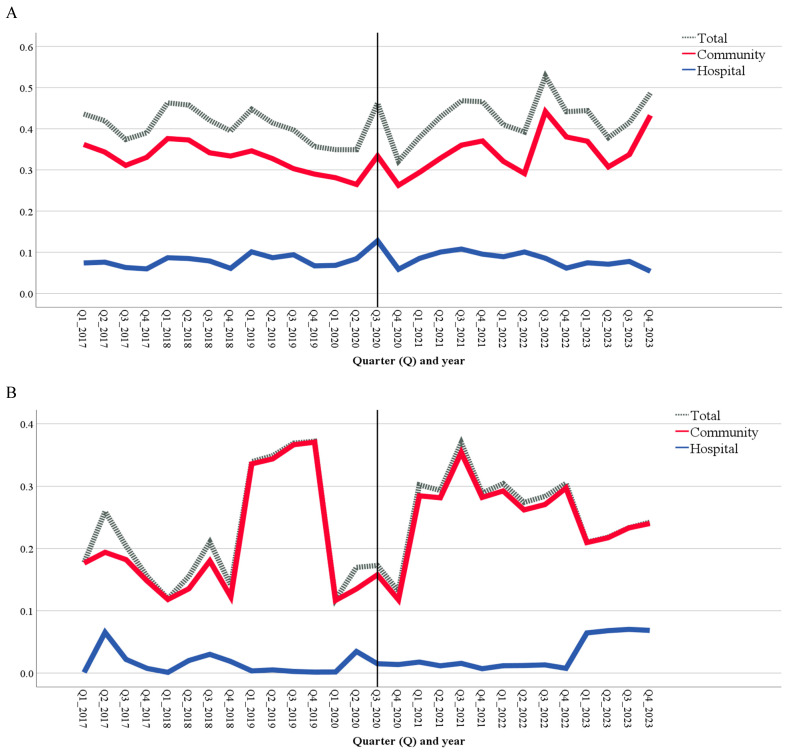
Consumption of systemic antifungals (**A**) and antiprotozoals (**B**), expressed in defined daily doses per 1000 inhabitants per day, disaggregated by quarter from 2017 to 2023. The black vertical line indicates the intervention point, marking the first wave of COVID-19 in the third quarter of 2020.

Figure 1A shows that the consumption of systemic antifungals peaked in the third quarter of 2020, coinciding with the first peak of COVID-19 in Kazakhstan [12]. This peak was observed in both the community and hospital sectors, resulting in an overall increase in total consumption. Another peak in community antifungal consumption occurred in the third quarter of 2022, while the hospital sector experienced a decline during the same period. Overall, the total consumption of systemic antifungals was influenced primarily by community consumption. With respect to systemic antiprotozoals, the peak in total and community consumption occurred in the fourth quarter of 2019 and the third quarter of 2021. Generally, the trends in total consumption closely mirrored those in community consumption (Figure 1B).

Table 4 presents the results of the interrupted time series analysis (ITSA) of the impact of the COVID-19 pandemic on the consumption of systemic antifungals. The term ‘intervention’ refers to the specific point in time when the COVID-19 outbreak began in Kazakhstan, specifically in the third quarter of 2020. This marks the key moment expected to influence drug consumption patterns due to the pandemic. The term ‘centering trend’ describes the method of adjusting the timeline so that this intervention point (the start of the pandemic) becomes the central reference for analysis. By centering the timeline around the intervention, it becomes easier to observe and compare trends in drug consumption before and after the COVID-19 outbreak, thereby isolating the pandemic’s effect on these trends. 

According to the ITSA, following the outbreak of COVID-19 in Kazakhstan in the third quarter of 2020, the total consumption of systemic antifungals increased by 0.038 defined daily doses per 1000 inhabitants per day (DID). During the same period, community consumption increased by 0.016 DID, and hospital consumption increased by 0.022 DID. The coefficients for the centering variable were estimated at −0.001, 0.000, and −0.001, respectively, reflecting trends in quarterly consumption rates over time.

Table 5 presents the results of the ITSA on the impact of the COVID-19 pandemic on the consumption of systemic antiprotozoals. The terms “intervention” and “centering trend” are used as defined in Table 4. Starting in the third quarter of 2020, the consumption rates of systemic antiprotozoals showed a negative trend, with total consumption decreasing by −0.016 DID, community consumption decreasing by −0.027 DID, and hospital consumption decreasing by −0.012 DID. The COVID-19 outbreak also negatively impacted the total consumption of metronidazole (−0.017 DID), whereas the consumption of secnidazole showed a very mild growth trend (0.001 DID).

Table 6 presents the projected trends for the consumption of systemic antifungals and antiprotozoals, both in total and within the community and hospital sectors, through 2030. Overall, the consumption of systemic antifungals is projected to remain stable until 2030. Similarly, the total and community consumption of antiprotozoals are also expected to remain stable. However, the consumption of systemic antifungals in the hospital sector is projected to decrease.

**Figure 2:**
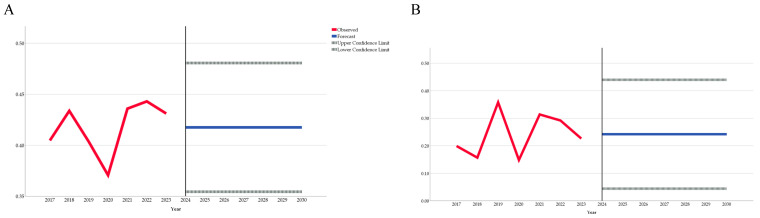

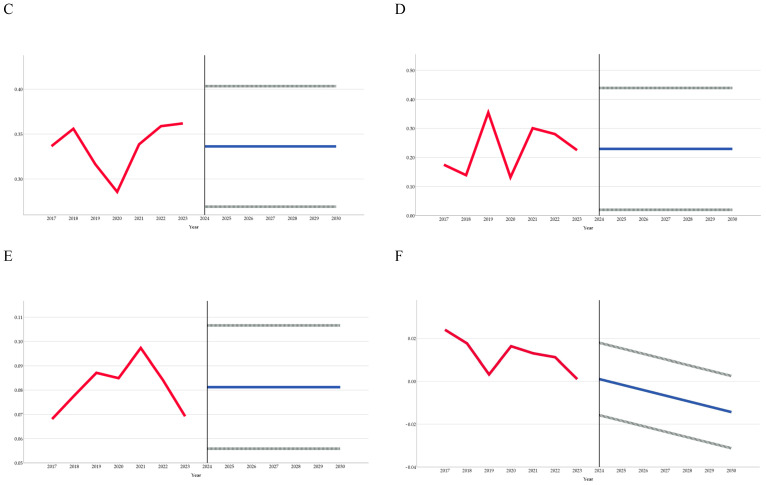
The observed and projected consumption rates of total antifungals (**A**), total antiprotozoals (**B**), community antifungals (**C**), community antiprotozoals (**D**), hospital antifungals (**E**), and hospital antiprotozoals (**F**) until 2030.

Figure 2 graphically illustrates the data from Table 6, depicting the observed and projected rates of consumption of systemic antifungals and antiprotozoals through 2030.

## 3. Discussion

This study aimed to evaluate the consumption patterns of systemic antifungals and antiprotozoals in Kazakhstan over a seven-year period, with a detailed analysis of both the community and hospital sectors. A particular emphasis was placed on investigating the impact of the COVID-19 pandemic on the consumption of these medications and conducting a forecast analysis up to 2030. From 2017 to 2023, an increasing trend in the consumption of antifungals and antiprotozoals was observed within the community sector. In the hospital sector, the consumption of antifungals has increased, whereas the consumption of antiprotozoals has decreased. The COVID-19 pandemic had a positive effect on the consumption of antifungals and a negative effect on the consumption of antiprotozoals. Forecast modeling indicates that the future trends in antifungal and antiprotozoal consumption until 2030 will largely remain stable, with the exception of antiprotozoal consumption in the hospital sector, which is projected to decline. These findings necessitate further discussion in the context of the literature to better understand the underlying factors driving these trends and to readjust the existing AMS program.

Antifungal stewardship is less often introduced and studied than antibiotic stewardship is, particularly in LMICs, where there is a paucity of consumption studies [2]. A recent point-prevalence study conducted in four hospital settings in India revealed that amphotericin B was the most commonly prescribed antifungal, accounting for 50.2% of all prescriptions, followed by fluconazole, which accounted for 31.6% [13]. Another study from a hospital setting in Chile also revealed that amphotericin B was the most consumed antifungal agent, followed by voriconazole [14]. In Lebanon, fluconazole is the most consumed antifungal agent, accounting for 20.99% of the total consumption [15]. In the community sector, fluconazole was the most consumed antifungal agent in Lebanon [15] and Tanzania [16]. A global study on antifungal consumption in 65 middle- and high-income countries revealed that fluconazole was the most commonly consumed systemic antifungal agent in high-income countries, whereas itraconazole was the most commonly consumed systemic antifungal agent in middle-income countries [17]. 

In this study, fluconazole was the most commonly consumed antifungal agent in both the hospital and community sectors, followed by ketoconazole, while amphotericin B accounted for less than 1% of the total. Over the period from 2017 to 2023, only the consumption of amphotericin B increased, as indicated by the positive AAPC, while the consumption of fluconazole and ketoconazole declined, as evidenced by negative AAPC values. However, the *p*-values for these trends in total consumption were not significant, possibly due to the short time frame (only 7 years). Another possible explanation for the insignificant *p*-value of the AAPC is the variability in consumption patterns that exist across different regions. Although this study did not focus on regional differences, future research should explore these variations to better understand the factors influencing antifungal use trends.

Among systemic antiprotozoals, nitroimidazole derivatives constitute the only group of antiprotozoal medications used in Kazakhstan. This group of medications is active against anaerobic bacteria and certain protozoal organisms, particularly those that cause trichomoniasis [18]. Metronidazole is the most consumed antimicrobial in some provinces of Uganda [19], and it is one of the most frequently used antimicrobials in Ghana [20] and Pakistan [21]. In Kazakhstan, metronidazole constituted the bulk of systemic antiprotozoal consumption, substantially surpassing the consumption of secnidazole. This might be attributed to better awareness among medical professionals about metronidazole, given its long-standing presence on the market, as well as the price difference, although secnidazole has a longer half-life and better tolerability [22].

In general, the consumption of both antifungals and antiprotozoals is driven by the incidence and type of infection, pathogen susceptibility patterns, existing standards of care, local prescribing practices, and potential drug toxicity [6]. For systemic antifungals, the provision of organ transplantation services is also an important driver [17]. Kazakhstan established its national heart, liver, and kidney transplantation programs in 2012 [23], but the rates of organ transplantation remain low [24,25], contributing to relatively low rates of systemic antifungal consumption. A large study investigating global rates of antifungal consumption reported a rate of 0.92 DID in 2018, which was higher in high-income countries than in middle-income countries [17]. The same study reported that antifungal consumption has experienced a decline in high-income countries and growth in middle-income countries [17]. In Kazakhstan, the overall rate of systemic antifungal consumption ranged between 0.37 and 0.44 DID, with an increase of 1.11%, confirming the observation that antifungal consumption is increasing in middle-income countries. The reasons behind this growth include the increased affordability of antifungals, particularly azoles; the growing population of immunocompromised individuals, particularly those living with HIV/AIDS; and a lack of appropriate infection preventive measures [17]. The extent to which these factors contribute to the increasing consumption of antifungals in Kazakhstan should be investigated in future studies.

The impact of the COVID-19 pandemic on the consumption of systemic antifungals was investigated in Turkey [26] and France [27], with significant growth reported in both countries. In France, this growth was particularly marked for voriconazole and caspofungin [27], whereas in Turkey, an increase in the consumption of amphotericin B was observed [26]. In Kazakhstan, the most notable growth was observed for ketoconazole, with an increase of 0.229 DID. The increase in ketoconazole consumption was accompanied by a decrease in fluconazole consumption in 2021, which may be attributed to supply chain disruptions caused by the COVID-19 pandemic. Kazakhstan has learned from these disruptions and has ambitious plans to develop its pharmaceutical industry, aiming for 50% of all medications consumed in Kazakhstan to be manufactured locally by 2025 [28].

This study also demonstrated that, following the onset of the COVID-19 pandemic in Kazakhstan, the growth in systemic antifungal consumption was more pronounced in the hospital sector than in the community sector (0.022 DID vs. 0.016 DID). This could be partly attributed to invasive fungal diseases in hospitalized COVID-19 patients, as secondary fungal infections are not uncommon in patients with a primary viral infection. However, not all antifungal administrations are appropriate or necessary. Furthermore, due to the shortage of essential laboratory tests during the pandemic, empirical antifungal use was common [29]. This highlights the need to implement antifungal stewardship programs to ensure the appropriate use of antifungals.

This study has both strengths and limitations that need to be considered when interpreting its results. The major limitation is that it utilizes an aggregated nationwide dataset, making evaluations of regional variations impossible. In addition, the COVID-19 pandemic has had multiple effects on the economy and healthcare system, introducing confounding factors that are difficult to fully account for [30]. Furthermore, the seven-year period may not be sufficient to capture long-term trends in the consumption of antifungals and antiprotozoals. Predictive models are inherently based on assumptions, and future changes in healthcare policies, practices, and the emergence of new antifungal and antiprotozoal drugs are difficult to anticipate. Despite these limitations, the study had several strengths, including the availability of a large nationwide dataset and data from both the hospital and community sectors. The findings offer valuable insights into the development and implementation of targeted AMS programs in Kazakhstan.

## 4. Materials and Methods

### 4.1. Data Sources

This retrospective study primarily relied on two data sources, with the primary source being the database created and maintained by Vi-ORTIS (Almaty, Kazakhstan), a company specializing in pharmaceutical market research. Vi-ORTIS collects data for both the community and hospital sectors via various methods. For the community sector, data are obtained from pharmaceutical suppliers and community pharmacies across the country [31]. The collected data represent all pharmaceutical sales nationwide. To ensure accuracy, the data undergo a multilevel validation process that includes checking returns, transfers between pharmacies and suppliers, and other quality control measures [31]. For the hospital sector, data are collected from “SK-Pharmacia” (Astana, Kazakhstan), the sole supplier of pharmaceuticals to hospitals in Kazakhstan, which serves both public and private facilities [31]. Similar to the community sector, the hospital sector data include information on supplies, returns, and transfers of pharmaceuticals between the supplier and hospital facilities. The Vi-ORTIS database does not include any patient-level information; it only contains data on the quantities of pharmaceuticals sold and/or supplied [31]. The data provided by Vi-ORTIS are frequently used for academic research on pharmaceutical consumption in Kazakhstan [11].

The Vi-ORTIS database is updated monthly and can be aggregated on a monthly, quarterly, and annual basis. The data encompass the entire healthcare sector, as well as specific community and hospital sectors. For this study, data on antifungals for systemic use (J02 code of the ATC) and on antiprotozoals for systemic use (P01 ATC code) [32] were extracted on 1 March 2024, for Kazakhstan, covering the period from 1 January 2017 to 31 December 2023. Systemic antifungals are classified as antibiotics (J02AA), imidazole derivatives (J02AB), triazole and tetrazole derivatives (J02AC), or other antifungals for systemic use (J02AX) [32]. All systemic antiprotozoals available in Kazakhstan belong to the group of nitroimidazole derivatives (P01AB) [32].

Data were downloaded annually to analyze past and future consumption trends and quarterly to assess the impact of the COVID-19 pandemic on consumption. Information on systemic antifungals and antiprotozoals was extracted at ATC level 5 (ATC5) and included product name, active ingredient, dosage form, active ingredients per unit dose, route of administration, number of tablets/capsules/suspensions/ampoules/vials per package, and the number of packages sold.

In addition to the Vi-ORTIS database, demographic yearbooks issued by the Bureau of National Statistics [33] were consulted to retrieve information on the population size in Kazakhstan during the study period.

### 4.2. Study Units

Consumption rates were assessed in defined daily doses (DDDs), a statistical measure of drug consumption defined by the World Health Organization (WHO) as the assumed average maintenance dose per day for a drug’s main indication [34]. DDDs per 1000 inhabitants per day (DID) enables standardization and comparison of drug usage across different populations and time periods. To calculate the DID, the Excel template developed by the Global Antimicrobial Resistance and Use Surveillance System (GLASS-AMC) was utilized [35].

The GLASS template is organized into separate spreadsheets with sections for entering the total amount of each drug consumed, population size, and time period of data collection. This template aligns with the ATC classification system and includes predefined formulas to convert the total amount of drugs consumed into DDDs and then calculate the DID. Throughout the process of data entry and analysis, the GLASS Manual on the Management of Antimicrobial Consumption, developed by the WHO, was consulted to ensure the accurate documentation and reporting of the findings [36].

### 4.3. Data Analysis

Since the consumption of systemic antifungals and antiprotozoals was obtained at equal time intervals, various time series analyses were employed on the calculated DIDs. Past trends in the consumption of systemic antifungals and antiprotozoals were evaluated on an annual basis via the average annual percent change (AAPC) approach, which provides a summary measure of the annual rate of change over a specified period [37]—7 years in this case (from 2017 to 2023). For each AAPC, 95% confidence intervals (95% CIs) were computed to quantify the precision of the estimate, and probability levels (*p* values) were calculated to assess the statistical significance of the observed trends.

To further investigate the impact of the COVID-19 pandemic on drug consumption, ITSA was utilized by means of autoregressive integrated moving average (ARIMA) models. ITSA allowed for the evaluation of changes in the level and trend in consumption rates before and after the onset of the pandemic. Calculations of DIDs were carried out on a quarterly basis for J02 and P01 in total, as well as separately for the community and hospital sectors. In addition, these calculations were performed for different ATC5 categories, and only those ATC5 categories of antifungals with a significance level of less than 0.05 were included in the final results. 

The concepts of ‘intervention’ and ‘centering trend’ were computed to assess consumption trends before and after the COVID-19 pandemic. The term ‘intervention’ referred to the specific point in time when a significant event occurred that was expected to affect the outcome being studied. In this analysis, the intervention was defined as the onset of the COVID-19 pandemic in Kazakhstan, specifically in the third quarter of 2020, when the first significant escalation of COVID-19 cases was observed [12]. This point served as the key moment when the impact of the pandemic on drug consumption was anticipated to begin. The term ‘centering trend’ referred to the adjustment of the data timeline so that the intervention point (in this case, the third quarter of 2020) became the central reference point in the analysis. Essentially, this involved re-aligning the data timeline to compare the period before the intervention directly with the period after it. This approach enabled a clearer comparison of trends and made it easier to assess the impact of the intervention on the data [38].

Predictive modeling was conducted to forecast future trends in the consumption of systemic antifungals and antiprotozoals in total, as well as in the community and hospital sectors up to the year 2030. The Expert Modeler function was employed to identify the best-fit epidemiological model for this purpose. Statistical significance for all analyses was determined with a *p*-value threshold of less than 0.05. The results of these analyses were visualized through graphical representations, including sequence and forecast charts. All the statistical analyses were conducted via SPSS version 26 (Armonk, NY, USA).

The minimal dataset used for this study is available in Supplementary Materials.

### 4.4. Ethics Statement

The study protocol (submission 802/23112023) was reviewed by the Nazarbayev University Institutional Research Ethics Committee (NU-IREC), which granted exempt status on 1 December 2023.

## Figures and Tables

**Table 1:** Total consumption of systemic antifungals and antiprotozoals, expressed in defined daily doses per 1000 inhabitants per day.

ATC5 * Code	Substance	Year							AAPC ⁰ (95% CI ^∞^, *p* Value)
		2017 DID ** (%)	2018 DID (%)	2019 DID (%)	2020 DID (%)	2021 DID (%)	2022 DID (%)	2023 DID (%)	
Antifungals									
J02AA01	Amphotericin B	0.00000 (0.00)	0.00000 (0.00)	0.00000 (0.00)	0.00004 (0.01)	0.00005 (0.01)	0.00003 (0.01)	0.00006 (0.01)	7.67 (−36.46; 82.43, *p* = 0.305)
J02AB02	Ketoconazole	0.04587 (11.34)	0.03639 (8.39)	0.04209 (10.43)	0.00002 (0.00)	0.38267 (87.80)	0.00001 (0.00)	0.00005 (0.01)	−71.09 (−95.77; 97.56, *p* = 0.079)
J02AC01	Fluconazole	0.31182 (77.07)	0.34600 (79.78)	0.31364 (77.72)	0.33093 (89.30)	0.05240 (12.02)	0.39380 (88.90)	0.36794 (85.36)	−3.63 (−33.96; 40.65, *p* = 0.406)
J02AC02	Itraconazole	0.04646 (11.48)	0.05076 (11.70)	0.04732 (11.73)	0.03911 (10.55)	0.00025 (0.06)	0.04843 (10.93)	0.06195 (14.37)	−14.72 (−69.96; 142.06, *p* = 0.355)
J02AC03	Voriconazole	0.00005 (0.01)	0.00004 (0.01)	0.00012 (0.03)	0.00007 (0.02)	0.00000 (0.00)	0.00023 (0.05)	0.00040 (0.09)	10.23 (−73.87; 364.95, *p* = 0.434)
J02AC04	Posaconazole	0.00000 (0.00)	0.00000 (0.00)	0.00000 (0.00)	0.00000 (0.00)	0.00036 (0.08)	0.00000 (0.00)	0.00000 (0.00)	34.45 (−46.32; 236.74, *p* = 0.223)
J02AX04	Caspofungin	0.00038 (0.09)	0.00042 (0.10)	0.00034 (0.08)	0.00038 (0.10)	0.00011 (0.02)	0.00042 (0.09)	0.00063 (0.15)	1.27 (−24.49; 35.83, *p* = 0.458)
J02AX05	Micafungin	0.00001 (0.00)	0.00006 (0.01)	0.00005 (0.01)	0.00003 (0.01)	0.00001 (0.00)	0.00002 (0.00)	0.00002 (0.01)	−6.68 (−31.52; 27.18, *p* = 0.295)
Total		0.40461 (100.00)	0.43367 (100.00)	0.40357 (100.00)	0.37058 (100.00)	0.43585 (100.00)	0.44295 (100.00)	0.43107 (100.00)	1.11 (−2.00; 4.32, *p* = 0.203)
Antiprotozoals									
P01AB01	Metronidazole	0.17174 (86.26)	0.12917 (82.40)	0.33527 (93.74)	0.12155 (82.03)	0.29249 (93.24)	0.27487 (94.23)	0.20822 (92.08)	7.22 (−12.10; 30.78, *p* = 0.204)
P01AB07	Secnidazole	0.02735 (13.74)	0.02759 (17.60)	0.02238 (6.26)	0.02663 (17.97)	0.02120 (6.76)	0.01683 (5.77)	0.01792 (7.92)	−7.93 (−12.58; −3.02, *p* = 0.005)
Total		0.19908 (100.0)	0.15676 (100.0)	0.35765 (100.0)	0.14818 (100.0)	0.31370 (100.0)	0.29170 (100.0)	0.22613 (100.0)	5.48 (−11.22; 25.32, *p* = 0.231)

* Anatomical Therapeutic Chemical Classification, level 5. ** Defined daily doses per 1000 inhabitants per day. ⁰ Average Annual Percent Change. ^∞^ 95% confidence interval.

**Table 2:** Consumption of systemic antifungals and antiprotozoals, expressed in defined daily doses per 1000 inhabitants per day in the community sector.

ATC5 * Code	Substance	Year							AAPC ⁰ (95% CI ^∞^, *p* Value)
		2017 DID ** (%)	2018 DID (%)	2019 DID (%)	2020 DID (%)	2021 DID (%)	2022 DID (%)	2023 DID (%)	
Antifungals									
J02AB02	Ketoconazole	0.03413 (10.14)	0.03054 (8.58)	0.02348 (7.42)	0.00002 (0.01)	0.28863 (85.25)	0.00001 (0.00)	0.00005 (0.02)	−69.46 (−95.23; 95.58, *p* = 0.081)
J02AC01	Fluconazole	0.26139 (77.67)	0.27958 (78.55)	0.24993 (78.98)	0.24909 (87.20)	0.04992 (14.75)	0.31267 (87.14)	0.30160 (83.35)	−3.36 (−31.48; 36.30, *p* = 0.404)
J02AC02	Itraconazole	0.04061 (12.07)	0.04580 (12.87)	0.04303 (13.60)	0.03654 (12.79)	0.00000 (0.00)	0.04611 (12.85)	0.06013 (16.62)	−25.98 (−89.07; 401.24, *p* = 0.351)
Total		0.33653 (100.00)	0.35592 (100.00)	0.31645 (100.00)	0.28565 (100.00)	0.33856 (100.00)	0.35880 (100.00)	0.36185 (100.00)	1.08 (−3.21; 5.56, *p* = 0.276)
Antiprotozoals									
P01AB01	Metronidazole	0.14781 (84.40)	0.11166 (80.23)	0.33231 (93.74)	0.10550 (79.97)	0.27961 (92.97)	0.26378 (94.02)	0.20726 (92.06)	9.58 (−12.15; 36.67, *p* = 0.168)
P01AB07	Secnidazole	0.02731 (15.60)	0.02752 (19.77)	0.02218 (6.26)	0.02642 (20.03)	0.02113 (7.03)	0.01678 (5.98)	0.01787 (7.94)	−7.92 (−12.54; −3.06, *p* = 0.005)
Total		0.17513 (100.00)	0.13918 (100.00)	0.35450 (100.00)	0.13191 (100.00)	0.30074 (100.00)	0.28056 (100.00)	0.22513 (100.00)	7.37 (−11.20 to 29.82, *p* = 0.190)

* Anatomical Therapeutic Chemical Classification, level 5. ** Defined daily doses per 1000 inhabitants per day. ⁰ Average Annual Percent Change. ^∞^ 95% confidence interval.

**Table 3:** Consumption of systemic antifungals and antiprotozoals, expressed in defined daily doses per 1000 inhabitants per day in the hospital sector.

ATC5 * Code	Substance	Year							AAPC ⁰ (95% CI ^∞^, *p* Value)
		2017 DID ** (%)	2018 DID (%)	2019 DID (%)	2020 DID (%)	2021 DID (%)	2022 DID (%)	2023 DID (%)	
Antifungals									
J02AA01	Amphotericin B	0.00000 (0.00)	0.00000 (0.00)	0.00000 (0.00)	0.00004 (0.05)	0.00005 (0.05)	0.00003 (0.04)	0.00006 (0.09)	7.67 (−36.46; 82.43, *p* = 0.305)
J02AB02	Ketoconazole	0.01175 (17.25)	0.00586 (7.53)	0.01860 (21.35)	0.00000 (0.00)	0.09404 (96.66)	0.00000 (0.00)	0.00000 (0.00)	-
J02AC01	Fluconazole	0.05044 (74.09)	0.06642 (85.43)	0.06372 (73.13)	0.08184 (96.37)	0.00247 (2.54)	0.08113 (96.41)	0.06634 (95.85)	−6.98 (−52.18; 80.93, *p* = 0.395)
J02AC02	Itraconazole	0.00585 (8.60)	0.00495 (6.37)	0.00429 (4.93)	0.00257 (3.03)	0.00025 (0.26)	0.00232 (2.76)	0.00182 (2.62)	−24.49 (−52.66; 20.44, *p* = 0.091)
J02AC03	Voriconazole	0.00003 (0.04)	0.00004 (0.05)	0.00012 (0.14)	0.00007 (0.08)	0.00000 (0.00)	0.00022 (0.26)	0.00034 (0.49)	15.08 (−72.17; 375.82, *p* = 0.405)
J02AX04	Caspofungin	0.00000 (0.00)	0.00042 (0.53)	0.00034 (0.39)	0.00037 (0.44)	0.00011 (0.11)	0.00042 (0.50)	0.00063 (0.91)	4.36 (−32.95; 62.44, *p* = 0.401)
J02AX05	Micafungin	0.00001 (0.02)	0.00006 (0.07)	0.00005 (0.05)	0.00003 (0.04)	0.00001 (0.01)	0.00002 (0.02)	0.00002 (0.03)	−6.68 (−31.52; 27.18, *p* = 0.295)
Total		0.06808(100.0)	0.07775 (100.0)	0.08712 (100.0)	0.08492 (100.0)	0.09729 (100.0)	0.08415 (100.0)	0.06921 (100.0)	1.14 (−5.42; 8.16, *p* = 0.341)
Antiprotozoals									
P01AB01	Metronidazole	0.02392 (99.87)	0.01750 (99.57)	0.00296 (93.78)	0.01605 (98.69)	0.01288 (99.42)	0.01109 (99.60)	0.00096 (95.88)	−27.75 (−55.90; 18.37, *p* = 0.076)
P01AB07	Secnidazole	0.00003 (0.13)	0.00008 (0.43)	0.00020 (6.22)	0.00021 (1.31)	0.00007 (0.58)	0.00004 (0.40)	0.00004 (4.12)	−4.30 (−35.62; 42.25, *p* = 0.394)
Total		0.02396 (100.0)	0.01758 (100.0)	0.00316 (100.0)	0.01626 (100.0)	0.01296 (100.0)	0.01113 (100.0)	0.00100 (100.0)	−27.59 (−55.30; 17.30, *p* = 0.073)

* Anatomical Therapeutic Chemical Classification, level 5. ** Defined daily doses per 1000 inhabitants per day. ⁰ Average Annual Percent Change. ^∞^ 95% confidence interval.

**Table 4:** Interrupted time series analysis of the impact of the COVID-19 pandemic on the consumption of systemic antifungals in Kazakhstan, expressed in defined daily doses per 1000 inhabitants per day.

Consumption		Estimate (DID *)	SE **	*p* Value
Total antifungals	Intervention	0.038	0.035	0.296
	Centering trend	−0.001	0.002	0.683
Community antifungals	Intervention	0.016	0.034	0.646
	Centering trend	0.000	0.002	0.945
Hospital antifungals	Intervention	0.022	0.013	0.096
	Centering trend	−0.001	0.001	0.197
Total consumption of selected antifungals by ATC5 *** code				
J02AB02 (Ketoconazole)	Intervention	0.229	0.092	0.020
	Centering trend	−0.011	0.006	0.063
J02AC02 (Itraconazole)	Intervention	−0.035	0.013	0.016
	Centering trend	0.002	0.001	0.042
J02AC04 (Posaconazole)	Intervention	≤0.001	≤0.001	0.006
	Centering trend	≤0.001	≤0.001	0.004
J02AX04 (Caspofungin)	Intervention	≤0.001	≤0.001	0.006
	Centering trend	≤0.001	≤0.001	0.004
J02AX05 (Micafungin)	Intervention	≤0.001	≤0.001	0.034
	Centering trend	≤0.001	≤0.001	0.252

* DID—Defined daily doses per 1000 inhabitants per day. ** SE—standard error. *** ATC5—anatomical therapeutic chemical classification, level 5.

**Table 5:** Interrupted time series analysis of the impact of the COVID-19 pandemic on the consumption of systemic antiprotozoals in Kazakhstan, expressed in defined daily doses per 1000 inhabitants per day.

Consumption		Estimate (DID *)	SE **	*p* Value
Total antiprotozoals	Intervention	−0.016	0.061	0.795
	Centering trend	0.004	0.004	0.344
Community antiprotozoals	Intervention	−0.027	0.061	0.669
	Centering trend	0.005	0.004	0.216
Hospital antiprotozoals	Intervention	−0.012	0.017	0.489
	Centering trend	0.002	0.001	0.098
Total consumption of antiprotozoals by ATC5 *** code				
P01AB01 (Metronidazole)	Intervention	−0.017	0/062	0.783
	Centering trend	0.004	0.004	0.291
P01AB07 (Secnidazole)	Intervention	0.001	0.003	0.686
	Centering trend	−0.001	0.000	0.013

* DID—Defined daily doses per 1000 inhabitants per day. ** SE—standard error. *** ATC5—anatomical therapeutic chemical classification, level 5.

**Table 6:** Projected consumption rates of systemic antifungal and antiprotozoals in Kazakhstan, expressed in defined daily doses per 1000 inhabitants per day, until 2030.

Year	Antifungals, DID *			Antiprotozoals, DID		
	Total	Community	Hospital	Total	Community	Hospital
2024	0.41747	0.33625	0.08122	0.24189	0.22959	0.00101
2025	0.41747	0.33625	0.08122	0.24189	0.22959	−0.00156
2026	0.41747	0.33625	0.08122	0.24189	0.22959	−0.00413
2027	0.41747	0.33625	0.08122	0.24189	0.22959	−0.00670
2028	0.41747	0.33625	0.08122	0.24189	0.22959	−0.00927
2029	0.41747	0.33625	0.08122	0.24189	0.22959	−0.01184
2030	0.41747	0.33625	0.08122	0.24189	0.22959	−0.01441
Model parameters	ARIMA (0.0.0), *p* ≤ 0.001	ARIMA (0.0.0), *p* ≤ 0.001	ARIMA (0.0.0), *p* ≤ 0.001	ARIMA (0.0.0), *p* ≤ 0.001	ARIMA (0.0.0), *p* ≤ 0.001	Holt, *p* = 0.997

* Defined daily doses per 1000 inhabitants per day.

## Data Availability

The data presented in this study are provided as Supplementary Materials.

## Supplementary Materials

The following supporting information can be downloaded at: https://www.mdpi.com/article/10.3390/antibiotics13090857/s1, Minimal dataset.
